# Supplementary material for: Long-Read Transcriptome of Equine Bronchoalveolar Cells
Source: Genes (Basel). 2022 Sep 25;13(10):1722. doi: 10.3390/genes13101722 (PMC9602388; doi:10.3390/genes13101722)
Supplement: Supplementary file 1 [file genes-13-01722-s001.zip › TableS1 and supplementaryFigs.pdf]

**Sample #**	**1**	**2**
Health status	Severe equine asthma	No respiratory symptoms
Breed	Selle Français	Swiss Warmblood
Sex	Mare	Mare
Age (years)	21	12
HOARSI[1]	4	1
Weight (kg)	634	613
Clinical score (/23)[2]	9	0
Tracheal mucus score (/5)[3]	2	1
*BALF yield %**	40	48
BALF macrophages %	56.25	48.50
BALF lymphocytes %	18	47.25
BALF neutrophils %	25.75	2.25
BALF mast cells %	0	2
BALF eosinophils %	0	0

Table S1. Characteristics of the study animals. *HOARSI, Horse Owner Assessed Respiratory Signs Index; BALF, Bronchoalveolar Lavage Fluid* *BALF yield % = [(Volume saline instilled (mL) – Volume saline reaspirated (mL)) / Volume saline instilled (mL)] x 100

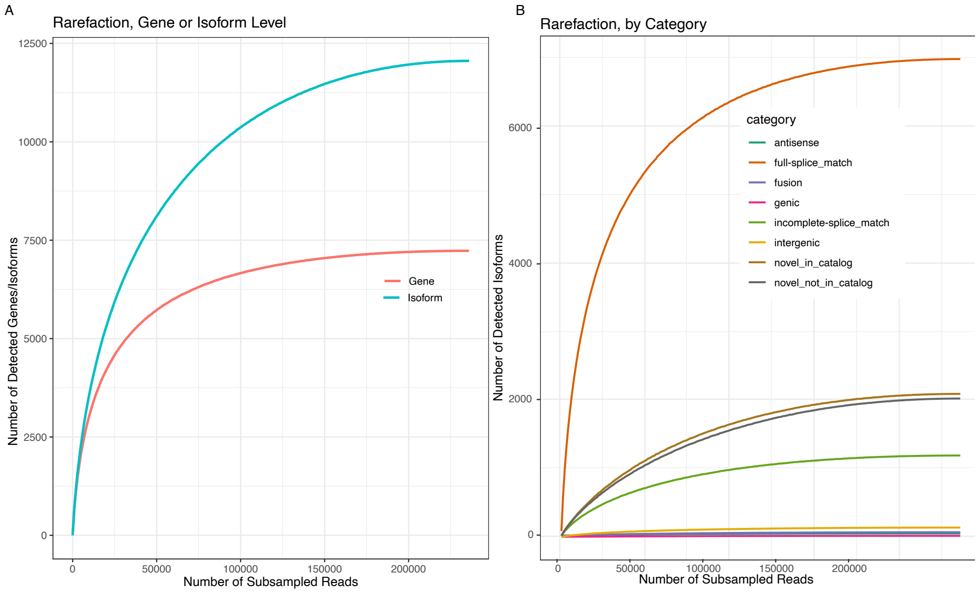

Fig S1. Saturation-discovery curve to determine the relationship between number of full-length reads subsampled and total number of genes and isoforms detected(A) and for different isoform structural classes(B).

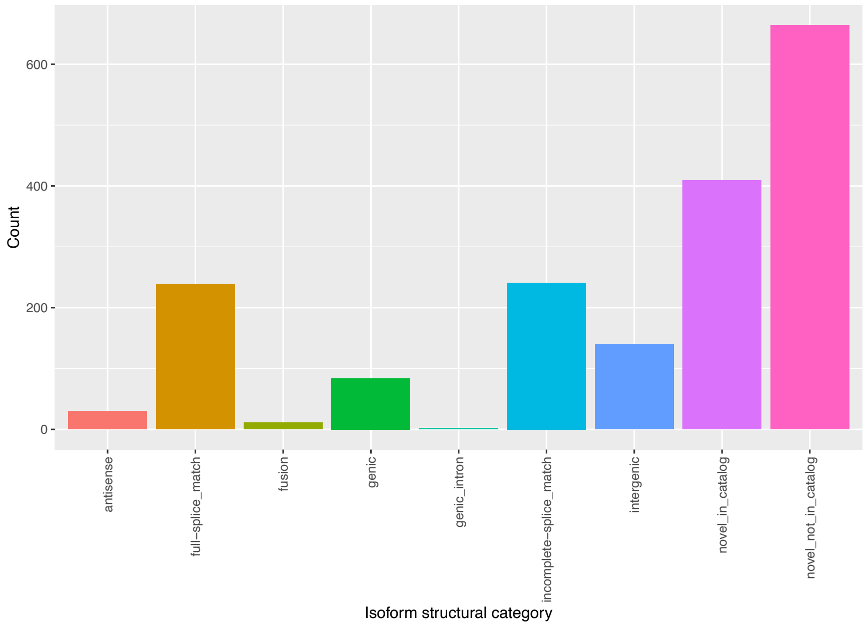

Fig S2: Count of filtered isoforms for different structural categories.

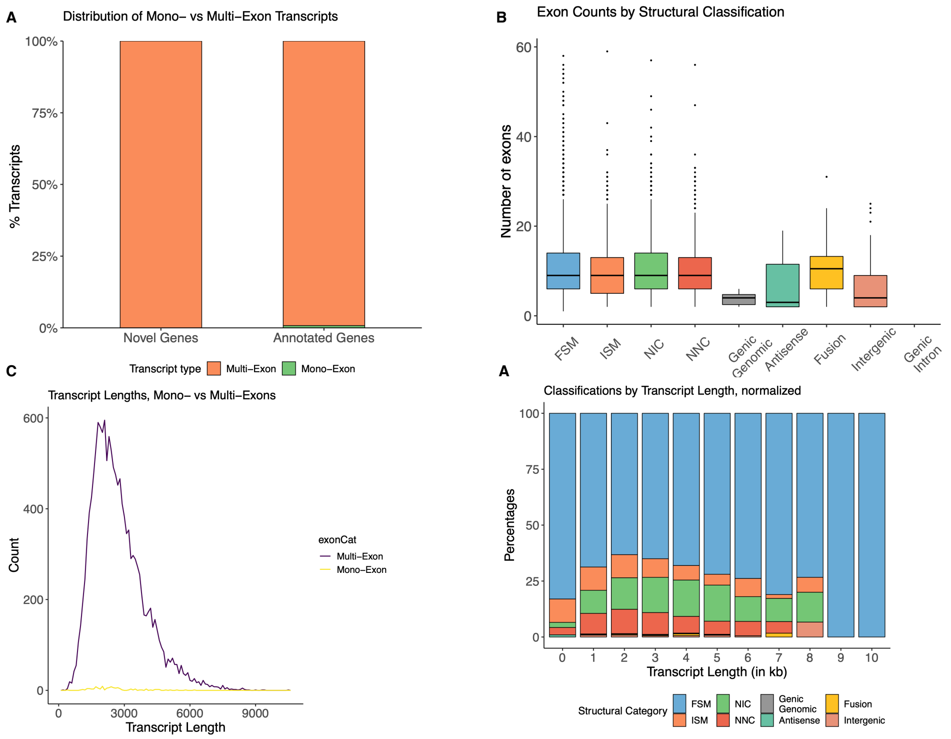

Fig S3: A. Exon distribution in novel and known genes. B. Exon counts in different structural category of transcripsts. C. Length of mono- and multi-exonic transcripts. D Length of transcripts classified by structural category

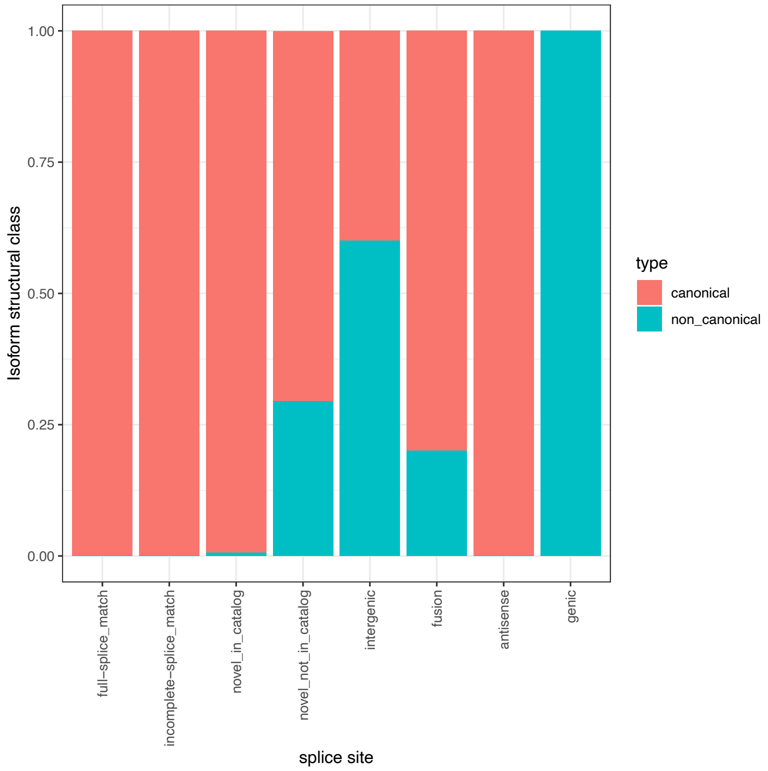

Fig S4: RT-Switching templates predicted by SQANTI3 in the different isoform classes.

References:
[1] Ramseyer A, Gaillard C, Burger D, et al. Effects of genetic and environmental factors on chronic lower airway disease in horses. Journal of Veterinary Internal Medicine. 2007;21:149–56
[2] Lavoie J-P, Bullone M, Rodrigues N, et al. Effect of different doses of inhaled ciclesonide on lung function, clinical signs related to airflow limitation and serum cortisol levels in horses with experimentally induced mild to severe airway obstruction. Equine veterinary journal. 2019;51:779–86.
[3] Gerber V, Straub R, Marti E, et al. Endoscopic scoring of mucus quantity and quality: observer and horse variance and relationship to inflammation, mucus viscoelasticity and volume. Equine veterinary journal. 2004;36:576–82.
